# Performance of pulse contour and pulse wave transit time-based continuous cardiac output analyses: clinical validation of two methods in Thai patients undergoing cardiac surgery

**DOI:** 10.1186/cc13332

**Published:** 2014-03-17

**Authors:** P Wacharasint, P Kunakorn, P Pankongsap

**Affiliations:** 1 Phramongkutklao Hospital, Bangkok, Thailand

## Introduction

The aim was to evaluate the performance of arterial pressure-based cardiac output (APCO) [1] and pulse wave transit time- based cardiac output (esCCO) [2] monitors in Thai patients undergoing coronary artery bypass graft surgery (CABG) with cardiopulmonary bypass.

## Methods

We studied 50 Thai surgical patients undergoing CABG with cardiopulmonary bypass and requiring pulmonary artery catheter and radial artery catheter placement as a standard of clinical care. All patients were measured for APCO using the Vigileo/FloTrac and for esCCO using the esCCO monitoring system. The data were compared with thermodilution cardiac output (TDCO) monitoring as a reference method, simultaneously at pre-induction, post-induction, and every 30 minutes thereafter until the completion of the surgery. The bias and precision were assessed using Bland-Altman analysis.

## Results

In total, 310 pairs of simultaneous measurements of APCO versus TDCO and 303 pairs of esCCO versus TDCO were obtained from 50 patients. Both APCO (*r *= 0.53, *P *< 0.0001) and esCCO values (*r *= 0.56, *P *< 0.0001) were correlated with TDCO values. Either of the changes in APCO (*r *= 0.63, *P *< 0.0001) or any changes in esCCO (*r *= 0.60, *P *< 0.0001) were correlated with changes in TDCO. For APCO relative to TDCO, the bias, precision, and the limits of agreement were 0.70, 1.63, and -2.5 to 3.9 l/minute, while those of esCCO were 1.20, 1.59, and -1.9 to 4.3 l/ minute, respectively. Comparisons of the bias of APCO and esCCO revealed a level of significance of *P *< 0.001.

## Conclusion

Despite the overestimation of cardiac output measurements, APCO and esCCO calibrated with patient information has shown an acceptable trend as compared with TDCO in Thai patients undergoing CABG with cardiopulmonary bypass. Compared with esCCO, APCO demonstrated no significant differences of precision; however, a lower mean bias was exhibited.
